# Ultra-Early Warning Signs of Mania in Participants of Bipolar Disorder Psychoeducation Groups: a Pilot Mapping Study

**DOI:** 10.1192/j.eurpsy.2026.11181

**Published:** 2026-07-31

**Authors:** G. Barilla’, C. Venco, M. Cattolico, S. Pacchioni, M. Melli, C. Soragna, G. Lucchini, D. Bussolotti

**Affiliations:** ASST Mantua, Department of Mental Health and Addictions, Mantua, Italy

## Abstract

**Introduction:**

Early relapse detection is pivotal in bipolar disorder (BD). Colom & Vieta’s group psychoeducation model teaches patients to identify personal prodromes and ultra-early warning signs (UEWS) that precede mania/hypomania by days or weeks (Colom & Vieta. *Psychoeducation Manual for Bipolar Disorder.* Cambridge Univ Press 2006). Each UEWS must be specific, subtle, easily recognisable and clearly different from baseline behaviour.

Early-sign training improves time to recurrence and reduces hospitalisation when added to pharmacotherapy (Morriss et al. *Cochrane Database Syst Rev* 2007). Our Mantua service has delivered this model since 2014, but UEWS content reported by our patients has not been systematically mapped.

**Objectives:**

To map UEWS of emerging mania reported by psychoeducated BD patients and explore patterns by sex and age.

**Methods:**

Two Colom-model bipolar psychoeducation groups in 2024 were included; all attendees were invited. Inclusion criteria: DSM-5 BD I/II, ≥ 2 manic or hypomanic episodes, ≥ 2 months euthymia. Twenty-five patients consented (mean age 47 ± 14 years; 13 women). A trained nurse led a ten-minute structured recall: “What is the first small change you notice before becoming ‘too high’?”. Two clinicians independently coded verbatim UEWS into nine a priori domains (sleep, appearance/self-care, activity, social/interpersonal, behaviour/media, risk/impulsivity, appetite, mood, cognition/functioning) adapted from the Manual and manic-prodrome research. Inter-rater κ = 0.86; discrepancies were resolved by consensus. Frequencies were tallied; χ^2^ tests compared domain distributions by sex and age (< 40, 40–59, ≥ 60); α = 0.05.

**Results:**

We identified 73 UEWS (median 3 per patient; range 1–6). Frequent domains were appearance/self-care (23 %), sleep/biological rhythm (17 %), activity/energy (16 %), behaviour/hobbies/media (14 %) and risk/impulsivity (11 %); other domains accounted for 19 %. Increased attention to appearance, waking slightly earlier, extra exercise, increased social-media posting and faster driving were the most common individual UEWS. Appearance/self-care UEWS were significantly more common in women than in men (68 % vs 32 %; χ^2^ = 4.7, p = 0.03). Patients < 40 years mentioned digital-media or music-related UEWS about twice as often as those ≥ 60 (χ^2^ = 4.2, p = 0.04), whereas older adults more often reported changes in food quantity.

**Conclusions:**

UEWS of mania in psychoeducated BD patients cluster into a limited set of recognisable domains and show sex- and age-related patterns. Integrating a concise nine-domain UEWS checklist into routine group psychoeducation may enable ultra-early, patient-led activation of behavioural strategies, clinician contact and pharmacological adjustment, shortening time to intervention beyond standard prodrome-based relapse prevention.

**Disclosure of Interest:**

None Declared

